# A step towards gender equity to strengthen the pharmaceutical workforce during COVID-19

**DOI:** 10.1186/s40545-020-00215-5

**Published:** 2020-05-15

**Authors:** Nadia Bukhari, Mehr Manzoor, Huma Rasheed, Bismah Nayyer, Madeeha Malik, Zaheer-Ud-Din Babar

**Affiliations:** 1 School of Pharmacy, University College London, London, WC1N 1AX UK; 2 Department of Health Policy and Management, Tulane University, New Orleans, LA USA; 3 Institute of Pharmaceutical Sciences, University of Veterinary and Animal Sciences, Lahore, Pakistan; 4 Department of Population Health Sciences, Faculty of life Sciences & Medicine, King’s College London, London, SE1 1UL UK; 5 Hamdard University, Institute of Pharmaceutical Sciences, Islamabad, Pakistan; 6 Department of Pharmacy, University of Huddersfield, Queensgate, Huddersfield, HD1 3DH UK

## Abstract

There is plenty of evidence to support that women leaders are needed in the health and pharmaceutical sectors, although most of the leadership positions in global health are predominantly occupied by men. This is a major challenge to global health policy. Gender diversity and inclusion within the pharmaceutical workforce is integral to optimal patient care. Women continue to be underrepresented in senior and leadership positions within pharmacy, despite outnumbering the men in the global pharmacy workforce. This commentary highlights the need towards gender equity and discusses the several key initiatives that are building momentum and making substantial progress towards this agenda in the pharmaceutical workforce.

## Background

Gender discrimination is deeply rooted into global work force practices. One example was Amazon hidden artificial intelligence recruiting system, which was showing prejudice towards women resumes. The system was closed down in 2018 after facing harsh criticism from the public [1]. Gender equality is integral for a sustainable future and having equal rights and opportunities is an absolute necessity for women and girls. The United Nations has identified a set of 17 Sustainable Development Goals (SDGs) and gender equality (SDG 5) is one of them. Achieving equality by 2030 requires urgent action [2]. Gender equity refers to the fairness of treatment for both genders according to their needs [3]. This may include equal treatment or treatment that is different; however, it is equivalent in terms of rights, obligations, and opportunities [3]. Gender equity is the means to achieving gender equality by correcting for the gender discriminations and biases that disproportionately affect one gender over the other, thus improving economic outcomes for all [4].

Women make up majority of the global health workforce. Within the United States, they contribute $3 trillion to the health care economy by making health decisions for themselves and for their families [5]. There is plethora of evidence to support why women are needed in healthcare and within the pharmaceutical workforce, yet, majority of the leadership positions within global health, insurance companies, and pharmaceutical companies are populated exclusively by men. This is vital as leaders play an important role on key organizational decisions such as hiring, promotion, salary and resource allocation that determine the gender composition of the workforce.

Crisis can be an opportunity for moving a step closer to gender equality. The responsibility of care for those infected, or for taking ownership of the disruption to routine life such as school closures, is likely to fall disproportionately onto women. As the majority of the health workforce are women, they are in the majority when it comes to being at the frontlines in the flight against COVID-19 In addition, the global workforce has moved online and predominantly working from home. COVID-19 has sparked an opportunity to have greater flexibility but also create gender-equality in the workplace.

## Delivered by women, led by men – a call for gender equity in the global health workforce

Gender inequalities in the health workforce lead to maldistribution of health workers in the formal and informal health workforces [6]. The need for a gender equal workforce has been well established. Yet gender gaps in global health workforce are glaring.

Despite making majority of the global health workforce, women are not represented in the leadership roles and decision-making positions [7]. These gender gaps highlight the vertical segregation within the field of global health: women deliver global health while men lead it [7]. A report by Global Health 50/50 shows that 69% of global health organizations are headed by men, and 80% of board chairs in global health are men. Only 20% of global health organizations were found to have gender parity on their boards, while 25% had gender parity at senior management level [﻿8﻿].

Women in global health are concentrated in underpaid and often unpaid jobs [6]. The World Economic Forum Global gender gap report 2018 estimates that the average gender pay gap by country to be around 16%. However, these gaps are higher and widespread in health and social care sectors with around 26% in high-income countries and 29% in upper middle-income countries [7]. Furthermore, examples of gender gaps within global health include workplace violence and sexual harassment and often these issues are underreported or unreported due to social stigma or fear of retaliation. Although both men and women may face violence or sexual harassment, women are disproportionately the victims of such cases. Often female health workers face sexual harassment from their male colleagues, male patients, and/or members of their community [6]. Violence and harassment harms women by limiting their ability to do their job, forcing them to quit their jobs, lowering their morale, and causing them emotional as well as physical distress [7].

Women are not only underrepresented in leadership roles, but also overconcentrated in certain medical fields such as nursing and midwifery. This presents a picture that global health jobs are also horizontally segregated by gender. The female-dominated fields such as nursing and midwifery are often associated with lower status, pay, and prestige [7]. Men, on the other hand, enjoy senior leadership roles, higher status, and higher pay scales. This is true even if men enter female-dominated jobs or roles as they are more likely to climb the leadership ladder as compared to their female colleagues [9].

## Global pharmaceutical workforce from a gender lens

The pharmaceutical workforce plays a vital role within health care systems by improving healthcare outcomes through providing first point of contact to the individuals, offering medical advice, and ensuring smooth supply of medicines [10]. Women continue to be underrepresented in senior and leadership positions within pharmacy, despite outnumbering men in the global pharmacy workforce [11]. The International Pharmaceutical Federation (FIP) predicts that by year 2030, more than 70% of the global pharmacy workforce would comprise of women [12]. This is a significant improvement for women in pharmacy, as by 1970 women only 9% of the women were in pharmaceutical workforce. However still there are very few women who are in leadership roles [13].

The research has shown that female pharmacists were found to be very committed to their work despite family commitments [14], however there are several barriers for women to progress within the pharmacy and the family and child-rearing responsibilities are among the top [13].

While there is increase in the female enrolment in pharmacy schools around the world, enrolment rates vary from country to country. For example, in Pakistan and Sri Lanka female enrolment in pharmacy is 50% [15] while Chile has lower rate of enrolment [16]. Certain fields also have lower rates of male enrolment such as in the Faculty of Life Sciences at a leading university in Pakistan, where the male enrolment was less than 20% [15]. Understanding factors affecting female participation in pharmaceutical education is key to achieving gender equity in the pharmaceutical workforce.

Another glaring policy issue within pharmaceutical workforce is the gender pay gap, which is attributed to direct discrimination against female pharmacists as well as factors such as lack of mentoring, absence of networking with colleagues, less effective negotiation behaviours, and failure of employers to comply with equal opportunity regulations [17].

There are country-wide differences in satisfaction rates among female pharmacists. For example, within USA, female pharmacists were reported to be more satisfied as compared to their male counterparts, despite heavy workload and job-related stress factors including lower salaries [15]. While in the UK female community pharmacists enjoy different aspects of their roles but their job satisfaction decreased due to their challenging and demanding work environments [18]. In Iran, male pharmacists were found to have higher job satisfaction levels due to higher job security, income levels, and job expectancy [19]. Within Pakistan, female pharmacists were more self-aware, empathic, possessed social skills, had better work-life balance and job satisfaction, and were found more emotionally intelligent as compared to their male colleagues [20]. However, factors such as workplace culture, workload, and social support were found to be integral to improving female pharmacists’ productivity and acceptance [21].

Furthermore, anecdotally, female pharmacists are rarely seen working at community pharmacies in most of the developing countries including Pakistan. This is coupled with the fact that the availability of qualified person in reality is not mandatory at these retail outlets along with lack of social and cultural support required to engage these female pharmacists at community pharmacies. This sector has a huge potential and opportunities to envisage females promoting small-scale business ventures led by pharmacists.

## A step towards gender equity in pharmaceutical workforce

Female pharmacists bring unique perspectives to the field. Improving gender equality within pharmaceutical workforce requires urgent action. Research shows that decent work environments, flexible work hours, day care centres, breast-feeding breaks and paid maternity leaves are essential policy tools to improve women’s inclusion in the workforce. Similar initiatives are needed in the pharmaceutical workforce. The role of mentors and sponsors has also found to be effective to increase female leadership. For example, to gain leadership positions the Global Agenda Council for Women Empowerment gives insights into the steps that can be instituted to improve female leadership, which among all steps includes mentorship, “Women do not move up into strategic roles because they are not sponsored into them” [22].

Thus, more inclusive job hiring strategies must be introduced to engage female pharmacy work force ensuring unbiased evaluation of resumes, evaluations on performance criteria and designing specific mentoring programs to train and encourage females to move up to leadership positions in order to ensure gender equity in pharmacy workforce. There are several key initiatives that are building momentum and making substantial progress towards this agenda in the pharmaceutical workforce. These include the following.

### The International Pharmaceutical Federation’s (FIP) - call to action #EquityRx – WDG10

Achieving gender equity in the pharmaceutical workforce is vital to the implementation of FIP’s Pharmaceutical Workforce Development Goals (PWDGs), particularly PWDG 10 (Gender and Diversity Balances) [23] PWDG10 calls for all countries to have clear strategies for addressing gender and diversity inequities in the pharmaceutical workforce, continued education and training, and career progression opportunities.

Some of the goal’s indicators and mechanisms include:

i. Demonstration of strategies to address the gender and diversity inequities across all pharmaceutical workforce and career development opportunities.
ii. Ensure full and effective participation and equal opportunities for leadership at all levels of decision-making in pharmaceutical environments; avoidable barriers to participation for all social categories are identified and addressed.
iii. Engagement and adoption of workforce development policies and enforceable legislation for the promotion of gender and diversity equity; policies and cultures for the empowerment of all without bias.

The International Pharmaceutical Federation (FIP) has made a commitment to support its members in working towards the Workforce Development Goals (WDGs) and has set in motion strategic mechanisms to meet this aim, including the establishment of the Workforce Development Hub (WDH) and more recently the FIP Workforce Transformation Programme (WTP) [23]. This is a policy framework and roadmap that provides guideline to promote gender equity in pharmacy.

### National alliance for women in pharmacy – Pakistan

Recognizing the lack of female representation in top positions within pharmaceutical workforce of Pakistan, and the need to provide networking opportunities for female pharmacists in Pakistan, National Alliance for Women in Pharmacy (NAWP) initiative was launched in 2019 under Pakistan Pharmacist Association. The continuous absence of females in the executive public and private meetings and decision-making forums was one of the key driving forces behind the establishment of this platform. The main vision of this initiative is to promote gender equity within Pakistan’s pharmaceutical workforce, empower women and promote female leadership in pharmacy. It is the first organization of its kind, within the pharmaceutical workforce of Pakistan.

Since its inception, NAWP has reached a membership of 1400+ female pharmacists in Pakistan. Under this platform, meaningful conversations have been curated to address career and professional developmental needs of the female pharmacists by understanding their unique perspectives, needs, expectations, aspirations, and contributions within the field. It also has been involved in examining women’s health care needs and challenges to women’s access to health systems within Pakistan. These insights have play an important role in ensuring the role female pharmacists can play in better serving the needs of their communities.

NAWP has also undertaken representation at global forums such as the International Pharmacy Congress and Exhibition by launching a seminar on “Gender Equity and Women Empowerment” and held an award distribution ceremony to recognize women’s contributions and leadership within the profession. Through these global as well as national partnerships, NAWP has been instrumental in advocating for the need for providing decent work environments for female pharmacists as well as providing mentorship opportunities.

Lack of women leaders and role models is often considered a barrier to gender equality within the workplace [7]. To address this gap, NAWP is on its way to start mentorship program through its international linkages and highlight the female role models by bringing more visibility to their stories and contributions. NAWP has already gained support global leaders in pharmacy and global health including the CEO of FIP and aligns with FIP’s #EquityRx call to action.

Recently, NAWP developed a 10-point guidance for pharmacy teams in Pakistan for safety precautions that need to be taken during theCOVID-19 pandemic; endorsed by the Ministry of National Health Services, Pakistan [24].

.

Addressing gender inequities in the pharmaceutical workforce requires deeper understanding of local cultural and social contexts to gain insights of challenges women face in the workforce. NAWP provides an example of how local activism and movement can pave the way to address the deep-rooted gender norms, stereotypes and roles that limit women’s career choices and create barriers to their career progression. It is a step towards gender equity in Pakistan’s pharmaceutical workforce and offers policy implications and learnings for other similar low-and-middle-income countries.
